# Protein signature characterizing *Helicobacter pylori* strains of patients with autoimmune atrophic gastritis, duodenal ulcer and gastric cancer

**DOI:** 10.1186/s13027-017-0133-x

**Published:** 2017-04-27

**Authors:** Valli De Re, Ombretta Repetto, Stefania Zanussi, Mariateresa Casarotto, Laura Caggiari, Vincenzo Canzonieri, Renato Cannizzaro

**Affiliations:** 1 Facility of Bio-Proteomics, Immunopathology and Cancer Biomarkers, IRCCS CRO National Cancer Institute, Via F. Gallini 2, 33081 Aviano, Italy; 2 Microbiology-Immunology and Virology, IRCCS CRO National Cancer Institute, Aviano, Italy; 3 Pathology Gastroenterology, IRCCS CRO National Cancer Institute, Aviano, Italy; 4 Gastroenterology, IRCCS CRO National Cancer Institute, Aviano, Italy

**Keywords:** Adenocarcinoma, Autoimmune atrophic gastritis, Comparative proteomics, DIGE, Duodenal ulcer, Gastric cancer, *Helicobacter pylori*

## Abstract

**Background:**

*Helicobacter pylori* (*H. pylori*) represents a key factor in the etiology of autoimmune atrophic gastritis (AAG), duodenal ulcer (DU) and gastric cancer (GC). The aim of this study was to characterize the differential protein expression of *H. pylori* isolated from gastric biopsies of patients affected by either AAG, DU or GC.

**Methods:**

The *H. pylori* strains were isolated from endoscopic biopsies from the stomach of patients with gastric disease. Protein profiles of *H. pylori* were compared by two-dimensional difference in gel electrophoresis (2D-DIGE) coupled with mass spectrometry (MS) for the identification of significantly different spots (Student *t*-test, *p* < 0.05).

**Results:**

A total of 47 differentially expressed spots were found between *H. pylori* isolated from patients with either DU or AAG diseases and those isolated from patients with GC (Anova < 0.05, log fold change >1.5). These spots corresponded to 35 unique proteins. The identity of 7 protein spots was validated after one-dimensional electrophoresis and MS/MS analyses of excised gel portions. In *H. pylori* isolated from DU-patients a significant increase in proteins with antioxidant activity emerged (AroQ, AspA, FldA, Icd, OorA and ScoB), together with a higher content of proteins counteracting the high acid environment (KatA and NapA). In *H. pylori* isolated from AAG-patients proteins neutralizing hydrogen concentrations through organic substance metabolic processes decreased (GroL, TrxB and Tuf). In addition, a reduction of bacterial motility (FlhA) was found to be associated with AAG-*H. pylori* isolates. In GC-*H. pylori* strains it was found an increase in nucleic acid-binding proteins (e.g. DnaG, Tuf, RpoA, RplU) which may be involved in a higher demand of DNA- and protein-related processes.

**Conclusion:**

Our data suggest the presence of specific protein signatures discriminating among *H. pylori* isolated from either AAG, DU or GC. Changes in protein expression profiles evaluated by DIGE succeeded in deciphering part of the molecular scenarios associated with the different *H. pylori*-related gastric diseases.

**Electronic supplementary material:**

The online version of this article (doi:10.1186/s13027-017-0133-x) contains supplementary material, which is available to authorized users.

## Background

*Helicobacter pylori* (*H. pylori*) is a class I bacterial pathogen (IARC) colonizing approximately 50% of the world’s population. The infection increases the risk of extragastric and gastric diseases, including duodenal ulcer (DU), autoimmune atrophic gastritis (AAG) and gastric cancer (GC) [1–4]. It is estimated that about 3% *H. pylori*-infected individuals will develop a GC with an increased risk of 3-6-fold compared with non-infected population [5, 6].

Many virulent *H. pylori* genes have been reported to have a role in clinical outcomes of infection, with a predominant involvement of the plasticity region and *cag* pathogenicity island genes in GC development [7–10]. However, the precise mechanisms for GC development by *H. pylori* infection are still not completely understood. Analysis of the *H. pylori* proteome offered valid tools to delineate post-translational modifications and the complexity of gene expression and regulation characterizing *H. pylori* protein profiles associated with a particular clinical outcome [11–13]. The aim of this study was to investigate the *H. pylori* proteome profile by two-dimensional difference in gel electrophoresis (2D-DIGE) coupled with mass spectrometry (MS) and bioinformatics in order to correlate some differential *H. pylori* proteins to the clinical outcomes of gastric diseases in an Italian population.

## Methods

### Bacterial strains and culture conditions

The study was approved by the Internal review board and ethical committee of the IRCCS CRO, and Italian National Cancer Institute (IRB-14-2013). The *H. pylori* strains were isolated from endoscopic bioptic samples from the stomach (corpus and/or antrum), as previously reported [14]. Briefly, the biopsies were cultured in *H. pylori* Selective Medium (Bio-Mèrieux, Rome, Italy), and incubated at 37 °C in a microaerophilic environment (Campygen Oxoid, Ltd., Basingstoke, Hampshire, England) until growth evidence for at least 13–14 days. Several sweeps of colonies, considered representative of the whole *H. pylori* population, were subcultured in agar-blood plates, and after 3 days of incubation were collected and stored at −80 °C in a microbial storage medium (Microbank; Pro-Lab Diagnostics, Richmond Hill, Canada). Strains were revitalized after a median of 9 months (range of 2–98 months) in *H. pylori* Selective Medium, expanded in Columbia sheep blood agar, and then used for proteome extraction. Bacterial DNA extraction and PCR on the virulence factor CagA gene were performed in *H. pylori* strains isolated from patients accordingly to Repetto et al. [14] and Fasciana et al. [15].

### Patient characteristics

Fresh human gastric biopsies were obtained after patient informed consent. Patients were considered *H. pylori*-infected if results from cultures and histologic examination of the biopsy stained by Giemsa and/or serology for *H. pylori* (*H. pylori* IgG ELISA kit, BIOHIT HealthCare, Helsinki, Finland) were positive. According to confirmed histological patient diagnosis, *H. pylori* positive isolates were divided into DU-*H. pylori* (*n* = 11); AAG-*H. pylori* (*n* = 5), and GC-*H. pylori* (*n* = 25). Tissue biopsies were further grouped based on their anatomic gastric localization (A = antrum and C = corpus). Data of patients from whom *H. pylori* had been isolated are summarized in Table 1 and Additional file 2: Table S1.

**Table 1:** Clinicopathological characteristic of patients affected by gastric cancer, from whom *Helicobacter pylori* strains were isolated

Variable	
Tumor classification (Lauren)	
intestinal type	8
diffuse type	4
mixed type	5
indeterminate type	3
not available	5
Location	
proximal	13
distal	9
linitis plastica	1
not available	2
Stage	
0	1
1	5
2	0
3	9
4	0
not classified/not available	10
Operation (type of resection)	
Tis	1
T1	7
T2	2
T3	4
T4	1
not classified/not available	10
Lymph node status	
N0	7
N1	2
N2	0
N3	7
not classified/not available	9
Gastropanel	Mean (±SD)
PGI	157.9 (±113.8)
PGII	26 (±14.1)
PGI/II-ratio	6.2 (±2.6)
G-17	16 (±9.9)

*GC* gastric cancer, *PGI* mean of serum Pepsinogen I load, *PG2* mean of Pepsinogen II load, *G-17* Gastrin-17, *SD* Standard Deviation

### Protein labeling and DIGE

Proteins from frozen *H. pylori* cultures were extracted in methanol/chloroform, quantified and labeled as previously reported [14]. Prior to co-resolution on the same immobilized pH gradient (IPG) dry strip and two dimensional electrophoresis (2DE) gel, 25 μg of two bacterial lysates from two different strains was differentially labeled with 100 pmol cyanine fluorescent dyes (Cy3 and Cy5, GE Healthcare) and mixed with the Cy2-labeled internal standard, as described previously [16]. Internal standard included equal amounts of all the samples (nr = 41) within the experiment for a total of 21 gels. A dye swapping strategy was adopted to avoid a dye labeling bias. First dimensional isoelectric focusing (IEF) was carried out on 11-cm IPG strips (IPG pH 3 to 10 Bio-Rad, Milan, Italy) with Protean® IEF unit. The second dimension was performed using pre-cast 12% gels on Criterion™ Cells (Bio-Rad, Milan, Italy). For preparative gels, 300 μg of unlabelled protein pooled from equal amounts of samples was used, and stained with the ProteoStain solution (Proteomics Consult, Kampenhout, Belgium). Proteome maps were imaged using a Typhoon 940™ laser scanner (GE Healthcare, Uppsala, Sweden) and analysed using the DeCyder software version 6.5 (GE Healthcare). The EDA module was used for multivariate analysis of protein expression data, derived from BVA, and it allowed getting information about the ‘principal component analysis, PCA’ and the pattern analysis. Student’s *t* test was performed to assess the statistical significance of differentially expressed proteins based on average spot volume ratio. Based on average spot volume ratio, spots for which relative expression changed at least 1.5-fold (increase or decrease) at 95% confidence level (Student *t*-test; *p* < 0.05) were considered to be significant.

### Protein identification by mass spectrometry

**Fig. 1:**
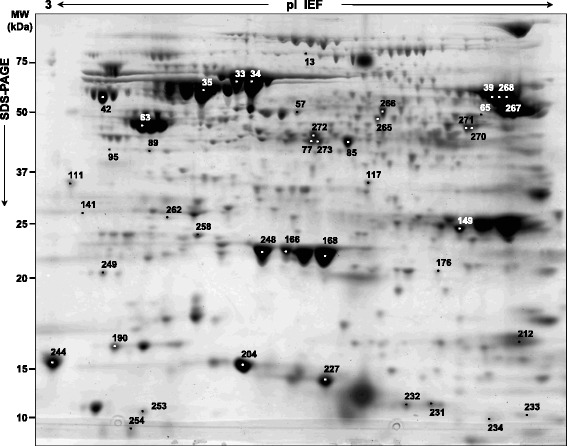
Representative micropreparative two-dimensional (2-D) protein map of Helicobacter pylori associated with duodenal ulcer (B). (A) Around 300 μg of DU-associated H. pylori unlabelled proteins were resolved by IEF over the pI range NL 3–10, followed by 8–16% gradient SDS-PAGE and stained. Numbered spots indicate the H. pylori differentially expressed proteins, which are listed in Table 2

Mass spectrometry analyses of differentially expressed spots were performed using either MALDI-TOF or LC-MS/MS. MALDI-TOF MS was performed on a Voyager-DE PRO Biospectrometry Workstation mass spectrometer (AB Sciex). While LC-MS/MS was performed using a LTQ XL-Orbitrap ETD equipped with a NanoEasy-HPLC (PROXEON, Thermo Fisher Scientific). Matched spots of interest were excised from the Coomassie Blue preparative gel, destained, trypsin-digested, and tryptic peptides were extracted by trifluoroacetic acid (TFA). In case of MALDI-TOF analyses, peptides were subjected to Zip Tip cleanup (Millipore, Milan, Italy), mixed with α-Cyano-4-hydroxycinnamic acid matrix solution (1:1, v:v) (LaserBio Labs, Sophia-Antipolis Cedex, France), and spotted on the MALDI target. The collected MALDI mass spectra were then processed by peptide mass fingerprinting (PMF) using Data Explorer (AB Sciex). Database searches were done with the MASCOT search engine version 2.3 (Matrix Science, London, UK), limiting the searches to bacterial proteins. Fig. 1 shows an example of a characteristics 2D gel map of an *H. pylori*-isolated strain with the indication of some of the identified proteins. To get an overview of the regulated proteins and their possible functional connections, the identified *H. pylori*-regulated proteins were analysed using the STRING tool (version 10; http://string-db.org) [17], after converting the protein accession numbers into ‘Kyoto Encyclopedia of Genes and Genomes, KEGG’ gene entries (http://www.genome.jp/kegg/). For each protein, KEGG pathways, biological processes and molecular functions were analysed according to the Gene Ontology (GO) description.

### Validation of the protein identified by using LC-MS/MS analysis

The correct identification of some proteins of interest was confirmed by searching them in gel portions of the corresponding MW after 12% 1DE. Pooled *H. pylori* protein extracts (15 μg per lane) were separated by 1DE, and images of ProteoStain-stained gel were acquired with the Typhoon Trio 9400™ laser scanner. Gel portions corresponding to the MW of around 52 kDa (Fig. 1, nr1, nr2), 22 kDa (Fig. 1, nr3) 15 kDa (Fig. 1, nr4), 12 kDa (Fig. 1, nr5, nr6), and 10 kDa (Fig. 1, nr7, nr8), were cut, reduced by incubation with 10 mM dithiothreitol (1 h at 57 °C), and alkylated with 55 mM iodoacetamide (45 min at room temperature). Samples were further washed with NH_4_HCO_3_, dehydrated, trypsin digested and processed for LC-MS/MS analyses.

## Results

### Proteomics analysis of *H. pylori* strains

*H. pylori* strains isolated from gastric biopsies of patients affected by either AAG, DU or GC were analyzed using the 2D-DIGE approach according to the tissue provenance of the *H. pylori* strains. *H. pylori* samples were obtained from 31 patients (Additional file 2: Table S1). Samples were obtained from 14 men and 17 women, with a mean age of 63.4 years (patients with GC) and 48.9 years (patients without GC). Table 1 shows the clinicopathological characteristics of GC-affected patients, from whom *H. pylori* strains were isolated. All the *H. pylori* strains isolated from both GC and DU patients were CagA+, while 2 strains isolated from 4 AAG patients resulted CagA+.

Firstly, we excluded that differences in protein abundance were dependent on the anatomical site from which *H. pylori* had been isolated (corpus and antrum). Protein profiles of *H. pylori* isolated from corpus were thus compared with those isolated from antrum biopsies. The stomach region resulted not to be a parameter significantly influencing the pattern of *H. pylori* protein expression (data not shown). Therefore, we continued our analyses independently on corpus or antrum sites of *H. pylori* isolation, and compared single maps per patient.

Comparative proteome analysis of *H. pylori* strains identified: (i) 29 significantly differentially expressed spots between *H. pylori* isolated from DU compared with those isolated from GC biopsies, with a fold difference ranging from +3.25 to−2.4, and (ii) 18 significantly differentially expressed spots between *H. pylori* strains isolated from AAG compared with those isolated from GC biopsies, with a fold difference ranging from 9.31 to−6.58 (Table 2). Details of protein identifications are shown in Table 2.

**Table 2:** Differentially expressed proteins of *Helicobacter pylori* related to autoimmune atrophic gastritis (AAG), duodenal ulcer (DU) or gastric cancer (GC)

ID^(a)^	Fold △^(b)^	Spot nr.	MW Da/pI	Accession number^(c)^	Protein name, gene name^(d)^	*H. pylori* strain	Score	Sequence Coverage	*p*-value	MS
AAG	9.31	13	27557/9.68	gi|238057731	tRNA pseudouridine synthase A, *truA*	*H. pylori* P12	32	26%	0.0024	MALDI-TOF
AAG	7.15	254	6644/10.96	gi|226703094	50S ribosomal protein L30, *rpmD*	*Leptothrix cholodnii*	37	30%	0.0045	MALDI-TOF
AAG	4.75	168	22335/5.88	gi|2507172	Probable peroxiredoxin or 26 kDa antigen, *trxB*	*H. pylori*	82	52%	0.00072	MALDI-TOF
DU	3.25	212	30558/6.6	O25984	4-diphosphocytidyl-2-C-methyl-D-erythritol kinase, *ispE*	*H. pylori*	32	11%	0.043	MALDI-TOF
DU	3.13	227	13411/6.1	B2UW13	10 kDa chaperonin or GroES protein, *groS*	*H. pylori* Shi470	80	42%	3.71E10-3	MALDI-TOF
DU	2.92	77	41655/5.92	gi|15611604	2-oxoglutarate-acceptor oxidoreductase subunit, *oorA*	*H. pylori* P12	216	31%	3.42E-03	LC-MS/MS
DU	2.92	273	42658/6.04	METK_HELPG	S-adenosylmethionine synthase, *metK*	*H. pylori* G27	112	31%	3.42E-03	LC-MS/MS
DU	2.75	149	28658/7.3	E1PY38	Putative heme iron utilization protein, *HPSJM 01705*	*H. pylori* SJM180	78	50%	6.27E10-3	MALDI-TOF
DU	2.67	204	16812/5.69	gi|560032	Neutrophil activating protein, *napA*	*H. pylori*	416	43%	0.029	LC-MS/MS
AAG	2.65	33	61816/5.64	gi|57014163	Urease β subunit, *ureB*	*H. pylori* J99	102	46%	0.014	MALDI-TOF
DU	2.49	85	38364/6.2	E1Q554	Aliphatic amidase, *amiE*	*H. pylori* PeCan4	80	31%	0.020	MALDI-TOF
AAG	2.48	57	67136/4.99	gi|226738136	Chaperone protein dnaK or Heat shock 70 kDa protein, *grpE*	*H. pylori* Shi470	32	8%	0.0075	MALDI-TOF
AAG	2.44	34	61846/5.64	gi|57014163	Urease β subunit, *ureB*	*H. pylori* P12	75	37%	0.0024	MALDI-TOF
DU	2.36	267	58700/8.6	B5Z7N5	Catalase, *katA*	*H. pylori* G27	43	24%	1.38E-03	MALDI-TOF
DU	2.36	268	58700/8.6	B5Z7N5	Catalase, *katA*	*H. pylori* G27	79	34%	1.38E-03	MALDI-TOF
AAG	2.22	166	22335/5.88	gi|2507172	Probable peroxiredoxin or 26 kDa antigen, *trxB*	*H. pylori* P12	97	48%	0.0013	MALDI-TOF
DU	2.21	258	22314/5.16	SCOB_HELPJ	Succinyl-CoA:3-ketoacid coenzyme A transferase subunit B, *scoB*	*H. pylori*	325	16%	8.92E10-3	LC-MS/MS
AAG	2.11	39	58706/8.70	gi|2493545	Catalase, *katA*	*H. pylori*	106	30%	0.0033	MALDI-TOF
DU	2.08	190	18528/5.1	B6JKY5	3-dehydroquinate dehydratase, *aroQ*	*H. pylori*	32	24%	4.37E-04	MALDI-TOF
DU	1.97	265	52416/6.48	gi|317181962	Aspartate ammonia-lyase, *aspA*	*H. pylori* F57	345	29%	1.76E-05	LC-MS/MS
DU	1.9	266	55022/6.50	gi|317009453	Leucyl aminopeptidase, *pepA*	*H. pylori* India7	283	25%	1.76E-08	LC-MS/MS
DU	1.9	249	14685/4.65	gi|122695106	Inorganic phyrophosphatase, *ppa*	*H. pylori*	425	34%	4.22E10-3	LC-MS/MS
DU	1.8	168	22335/5.9	P21762	Probable peroxiredoxin, *tsaA*	*H. pylori*	48	56%	0.02	MALDI-TOF
DU	1.75	271	47660/6.7	Q9ZN36	Isocitrate dehydrogenase, *icd*	*H. pylori* J99	70	30%	1.76E-03	MALDI-TOF
DU	1.75	270	47660/6.7	Q9ZN36	Isocitrate dehydrogenase, *icd*	*H. pylori* J99	49	8%	1.76E-05	MALDI-TOF
DU	1.69	253	12018/5.16	THIO HELPJ	Thioredoxin, *trxA*	*H. pylori* J99	1159	56%	1.24E10-3	LC-MS/MS
DU	1.64	176	22518/7.57	gi|298735719	Orotate phosphoribosyltransferase, *pyrE*	*H. pylori* B8	130	13%	1.59E-03	LC-MS/MS
AAG	1.62	65	20180/5.84	gi|208432952	Peptidoglycan-associated lipoprotein, *pal*	*H. pylori* G27	41	17%	0.027	MALDI-TOF
DU	1.6	244	17495/4.45	gi|108563525	Flavodoxin, *fldA*	*H. pylori* HPAG1	1590	79%	4.85E-05	LC-MS/MS
DU	1.5	272	41575/5.98	gi|317012405	2-oxoglutarate-acceptor oxidoreductase subunit, *oorA*	*H. pylori* Lithuania75	173	33%	7.33E–03	LC-MS/MS
AAG	1.47	77	74074/5.11	gi|12230111	Flagellar hook-associated protein 2, *fliD*	*H. pylori* J99	39	10%	0.045	MALDI-TOF
AAG	1.42	111	67838/6.17	gi|1706274	Bifunctional enzyme cysN/cysC, *cysN*	*Mycobacterium tuberculosis*	44	14%	0.073	MALDI-TOF
DU	−1.57	233	11804/10.25	RL21 HELHP	50S ribosomal protein L21, *rplU*	*H. hepaticus* ATCC 51449	61	9%	0.026	LC-MS/MS
DU	−1.65	95	38570/5.0	Q9ZJT5	DNA-directed RNA polymerase subunit alpha, *rpoA*	*H. pylori* J99	35	18%	3.26E-05	MALDI-TOF
AAG	−1.68	63	43734/5.17	gi|2494256	Elongation factor Tu, *tuf*	*H. pylori*	79	33%	9.00E-05	MALDI-TOF
DU	−1.7	262	25909/5.27	gi|15611222	Transcriptional regulator, *jhp 0381*	*H. pylori* J99	312	36%	0.049	LC-MS/MS
DU	−1.73	234	10402/9.37	DBH HELPJ	DNA-binding protein HU, *hup HP 0835*	*H. pylori* J99	52	11%	0.040	LC-MS/MS
DU	−1.79	231	13411/6.12	CH10 HELPS	10 kDa chaperonin, *groS*	*H. pylori* Shi470	53	25%	8.96E10–3	LC-MS/MS
DU	−2.1	232	49972/6.6	Q1CTD7	Ribosomal protein S12 methylthiotransferase, *rimO*	*H. pylori* HPAG1	32	13%	2.43E–03	MALDI-TOF
DU	−2.39	141	63933/8.9	E1Q0U8	DNA primase, *dnaG*	*H. pylori* SJM180	45	17%	9.87E–05	MALDI-TOF
DU	–2.4	89	43734/5.2	P56003	Elongation factor Tu, *tuf*	*H. pylori*	87	51%	8.57E–06	MALDI-TOF
AAG	−2.43	40	55280/5.29	gi|226739893	ATP synthase subunit alpha or F-ATPase subunit alpha, *atpA*	*H. pylori*	140	32%	0.0034	MALDI-TOF
AAG	−2.44	42	53252/6.04	gi|60392282	Flagellin A, *flhA*	*H. pylori* J99	88	30%	0.0098	MALDI-TOF
AAG	−2.48	141	63933/8.9	E1Q0U8	DNA primase, *dnaG*	*H. pylori* SJM180	45	17%	0.0089	MALDI-TOF
AAG	−2.9	89	43734/5.17	gi|2494256	Elongation factor Tu, *tuf*	*H. pylori*	90	33%	0.00013	MALDI-TOF
AAG	−5.35	248	22335/5.88	gi|2507172	Probable peroxiredoxin or 26 kDa antigen, *trxB*	*H. pylori*	44	34%	0.00095	MALDI-TOF
AAG	−6.58	35	58321/5.44	gi|226704136	60 kDa chaperonin, *GroL*	*H. pylori* P12	68	23%	0.0011	MALDI-TOF

^(a)^DU: differential spots of *H. pylori* isolated from DU *versus* GC (fold △values >1.5 or <1.5 indicate increase or decrease in content, respectively, in DU-*H. pylori*); AAG: differential spots of *H. pylori* isolated from AAG *versus* GC (fold △values >1.5 or <1.5 indicate increase or decrease in content, respectively, in AAG-*H. pylori*); ^(b)^fold difference calculated as log standardized abundance fold change; ^(c)^accession number of NCNInr or SwissProt databases;^(d)^gene names released by HUGO Gene Nomenclature Committee, or gene names adopted by STRING when different from HUGO ones

When it was not possible to identify spots as proteins belonging to *H. pylori* strains by MALDI-TOF and PMF, the analysis was performed by LC-MS/MS. Some proteins were present in more than one spot: for example, (i) the 2-oxoglutarate-acceptor oxidoreductase subunit (spots 77 and 272); (ii) the isocitrate dehydrogenase (spots 271 and 270); and (iii) the catalase (spots 268 and 267).

**Fig. 2:**
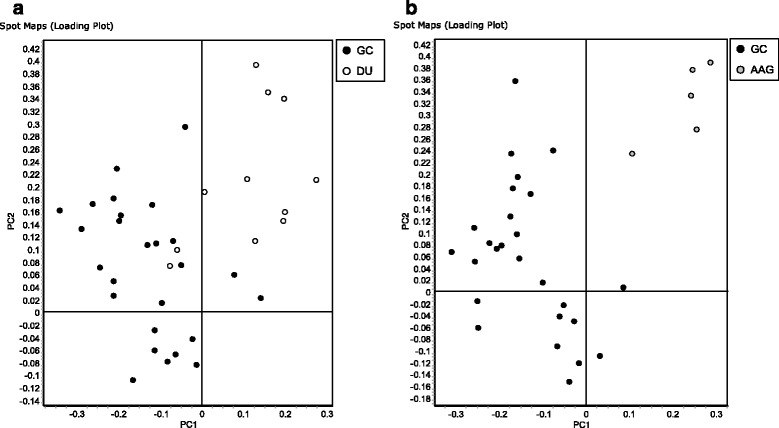
Principal component analysis of proteome maps of Helicobacter pylori isolates related to autoimmune atrophic gastritis (AAG), duodenal ulcer (DU) and gastric cancer (GC). The loading plots show an overview of the H. pylori spot maps of GC *versus* DU (a) and GC *versus* AAG (b). Each circle represents a spot map. AAG, DU and GC associated H. pylori spot maps are displayed in *grey*, *white* and *black*, respectively

The PCA based on protein expression clearly separated *H. pylori* isolated from GC from those isolated from either DU or AAG, although there was a partial overlap between *H. pylori* isolated from patients affected by DU and GC (Fig. 2).

**Fig. 3:**
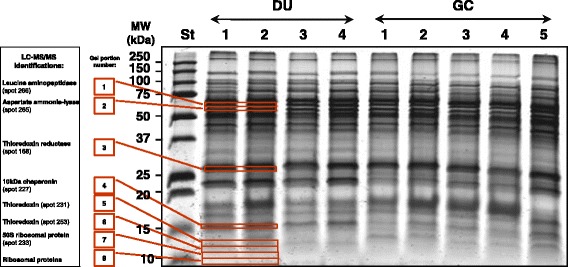
Protein separation by one-dimensional electrophoresis (1-PAGE) of Helicobacter pylori proteins extracted from duodenal ulcer (DU) or gastric cancer (GC) bioptic samples, and validation of the presence of some proteins. H. pylori protein extracts of either DU or GC biopsies were mixed and then aliquoted. After protein separation and gel staining, the 8 gel portions indicated by rectangles and numbers (nr.) were cut and processed for identification by mass spectrometry (LC-MS/MS)

### Validation of selected *H. pylori* proteins

For some proteins, to confirm the identity attribution by MS on 2D spots, their presence was searched by LC-MS/MS on 1D gel portions of the corresponding MW (Fig. 3). This approach allowed us to confirm the presence of leucine aminopeptidase (spot 266), aspartate ammonia-lyase (spot 265), peroxiredoxin 2 or thioredoxin reductase (spot 168), 10 kDa chaperonin (spot 227), thioredoxins (spots 231 and 253), and 50S ribosomal protein (spot 233). The presence of NapA protein (spot 204) was investigated in the 1DE band at around 15 kDa by MALDI-TOF MS: this band contained 4 masses (1180.4, 1340.49, 1826.74 and 2293.64 Da), which were also found in spot 204, and one additional (506.13 Da), which can be also achieved by analysis of the *in silico* digested NapA protein. These 5 masses allowed to identify NapA among the proteins at 15 kDa (Mascot results with peptide tolerance at 0.5 Da: Score 93; Expect 0.0054; Sequence coverage 48%). In order to exclude the presence of the found 5 peptide sequences in other proteins than NapA, the regions of similarity among other biological sequences were searched with Basic Local Alignment Search Tool (BLAST) (http://blast.ncbi.nlm.nih.gov/Blast.cgi). BLAST detected putative conserved domain of the ferritin-like superfamily and ferritin multi-domains, and confirmed the protein NapA at Max Score 248 (Query cover 100%; E value 2e–82; Identity 100%; Accession AAG28154.1) (Supplementary results, Additional file 1: Figure S1).

### Genetic interaction networks towards an understanding of *H. pylori* protein profiles

**Fig. 4:**
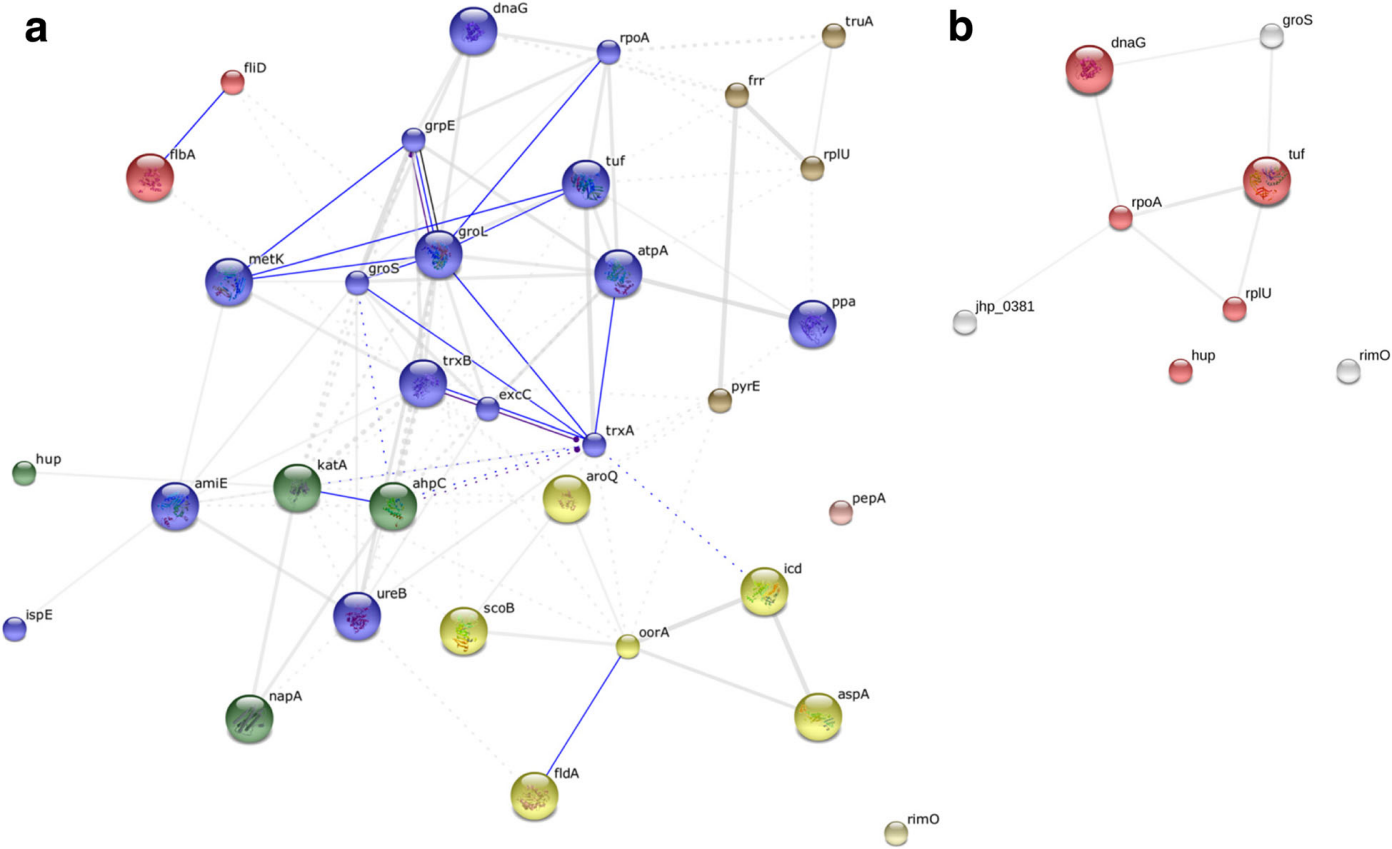
Protein-protein interaction maps of Helicobacter pylori proteins associated with gastric diseases. **a** The STRING tool (http://string-db.org) was used for making the networks with all the proteins listed in Table 2. Confidence views are shown, where the thickness of the connecting lines indicates the level of confidence. Stronger associations are represented by thicker lines. Different colors indicate different biological processes: (i) *blue* (organic substance metabolic process); (ii) *green* (defense against extreme environment conditions); (iii) *yellow* (oxidation reduction); (iv) *brown* (chemical reactions involving various nitrogenous compounds), and (v) *red* (bacteria motility). **b** The STRING tool was used for making the networks with the H. pylori proteins up-regulated in gastric cancer *versus* duodenal ulcer, which were mainly involved in nucleic acid binding (*red color*)

We used the STRING software matching the *H. pylori* strain 266995 to predict the protein-interactions based on the most differentially expressed proteins identified by 2D-DIGE analysis among *H. pylori* isolated from patients with either DU, AAG or GC. The obtained protein-protein interaction diagram (Fig. 4a, n. 33 proteins; *p*-value = 2.84e–10) revealed a widespread connectivity among these differentially expressed proteins with relevance to proteins involved in: (i) organic substance metabolic process (blue color); (ii) defense against extreme environment conditions (green color); (iii) oxidation reduction process (yellow color); (iv) chemical reactions involving various nitrogenous compounds (brown color), and (v) bacteria motility (red color). Two proteins (the leucyl aminopeptidase, *pepA*, and the ribosomal protein S12 methylthiotransferase methylthiotransferase, *rimO*) were not interactive with the other differentially expressed proteins. Both these proteins are presumably involved in the processing and regular turnover of intracellular proteins. The bifunctional enzyme cysN/cysC (spot 111; gi|1706274), involved in bacterial sulfate assimilation pathway, as well as the 50S ribosomal protein L30 (spot 254; gi|226703094), did not match with any *H. pylori* strains, the protein-interaction for these proteins thus remaining uncertain.

To better understand the network of *H. pylori* proteins associated with GC, we used the STRING software with the only up-regulated proteins found in GC-isolated *H. pylori.* This allowed us to evidence that the molecular pathway of these proteins was mainly related to nucleic acid binding (Fig. 4b; 7 proteins, *p*-value = 0.0311).

## Discussion

The scenario of molecular cross-talk between *H. pylori* and host gastric mucosa is finely regulated allowing a microbial persistence in the host, but also confers a risk for important diseases. Genomics and proteomics studies showed an high variability among *H. pylori* strains with strain-specific genes [18, 19] and proteins [20, 21] dispersed throughout the genome. In particular, DU-promoting gene cluster (*dupA* cluster) and *virB* gene forming a type IV secretory system (T4SS) have been proposed as a risk marker for both DU disease and the production of the pro-inflammatory IL-8 cytokine [22], while the intact *H. pylori cag-*PAI region has been associated with both DU and GC [23, 24]. The complexity of *H. pylori* proteome is further increased when *H. pylori* is analyzed in relation to gastric environment, in terms of both adaptation to hostile gastric conditions and host response(s) to disease(s) [25–27]. Furthermore, factors and molecular mechanisms linking *H. pylori* to GC are yet to be clearly elucidated.

The PCA analysis reported in our study showed a good discrimination of *H. pylori* classification based on patient’s disease, with the best result obtained analysing *H. pylori* isolated from patients with either GC or AAG, rather than *H. pylori* isolated from patients with DU. We compared by DIGE analysis paired groups of gastric disease (i.e. DU *versus* GC and AAG *versus* GC). The number of differentially expressed proteins of *H. pylori* isolates was higher when comparing DU *versus* GC (nr = 29) as compared with AAG *versus* GC (nr = 18), this result showing a more pronounced molecular diversity between *H. pylori* strains from GC and DU. This is in accord with the model of GC development, for which AAG, but not DU, is known to be a risk factor. Therefore, it is tempting to hypothesize that similarly *H. pylori* strains isolated from GC are more similar to *H. pylori* strains isolated from AAG than those isolated from DU.

It is well accepted that different microorganisms may have different possibility to regulate cell metabolisms. In our work, *H. pylori* isolated from patients with DU was found to regulate enzymes involved in metabolic pathways that could neutralize particularly high acid secretion of the gastric microenvironment (i.e. log fold increased expression; *ispE*: 3.25; *groS*: 3.13, *metK*: 2.92; *tuf*: 2.92, *amiE*: 2.49; Table 2; Fig. 4a). Different mechanisms allow the bacteria to proliferate in the highly acid gastric environment [28], and one of them involves ammonia generation from various substrates by enzymes such as urease (a nickel-containing enzyme composed of subunits UreA and UreB) [29] and amidases (i.g AmiE) [30]. Our work showed a higher content of AmiE and UreB in the proteome of *H. pylori* isolated from DU and AAG, respectively, as compared with GC. In particular, the AmiE enzyme is used as an alternative route for ammonia production necessary to maintain the pH homeostasis and to neutralize the gastric acidity [31], though ammonia may cause a direct tissue damage [32].

Diverse enzymes known to detoxify oxidants resulting from the high inflammatory status and to repair molecules [24, 33] have been found increased in content in DU-*H. pylori* proteome: i.e. two ‘catalases’ (spots 267, 268), which protect cells against reactive oxygen species through degradation of hydrogen peroxide to water and oxygen, and a ‘thioredoxin’ (spot 253), providing electrons to peroxiredoxins to remove reactive oxygen and nitrogen species [34]. Overall these *H. pylori* proteins were up-regulated in DU-*H. pylori* and may play a role in avoiding the higher acid and oxidative stress present in stomach microenvironment during DU with respect to that deriving from GC status. The reason for the oxidative stress behavior is that protein folding is severely affected by the gastric mucosa and inflammatory cells during DU.

Another protein strongly over-expressed in *H. pylori* isolated from patients with DU was the putative heme iron utilization protein, *H. pylori* SJM 01705 (spot 149). Iron must be acquired from the host, however, since an iron excess is toxic for bacteria, its acquisition is finely regulated by modulating the expression of this protein accordingly to the stomach conditions. In particular, this process may be particularly important in the case of *H. pylori* isolated from patients with DU, where due to stomach bleed, high levels of iron are present from hemoglobin degradation. Iron deficiency was shown to increase GC risk by increasing the virulence phenotype of CagA-positive *H. pylori* [35].

Another pathway upregulated in *H. pylori* isolated from patients with DU is involved in stress response with the up-regulated NapA and KatA proteins (spots 204 and 267, 268; Table 2; Fig. 4), which are both proteins known to protect *H. pylori* DNA from oxidative burst [36–38]. Moreover, NapA is also responsible for the recruitment of neutrophils to the site of infection, resulting in an increased influx of oxyradicals leading to collateral tissue damage [36], and since phagocytes are generally unable to kill the *H. pylori*, the production of NapA is perpetuated with the concomitant increase in tissue damage and katA production. In agreement with this model, it is noted that peptic ulcer was less frequent in children and this had been related with a lower number of neutrophils and CD3+ T-cells present in the gastric lamina propria of patients [39].

In the proteome of AAG-*H. pylori* compared with GC-*H. pylori*, the metabolic pathway neutralizing the gastric acid microenvironment was the most decreased, and indirectly it increased in GC-*H. pylori* (i.e. fold change *groL*:−6.58; *trxB*:−5.35; *tuf:−*2.9; *dnaG*:−2.48; *atpA:−*2.43; Table 2 and Fig. 4). Recently, Karlsson et al. [40] found an increase in levels of the acid response regulator ArsRS in *H. pylori* strain Nic25_A associated with intestinal metaplasia compared with another strain associated with DU. In the particular conditions of AAG, parietal cell antibodies and elevated levels of serum gastrin produced by the G cells of the antral gastric mucosa are typically found [41]. Under normal physiological conditions, gastrin acts on parietal cells to stimulate the secretion of gastric hydrochloric acid (HCL) and acidity in the gastric lumen inhibits its secretion by negative feed-back. While in AAG conditions, the immune system attacks the parietal cells leading to hypochloridia (low HCL), which results in a loss of negative-feedback on gastrin secretion. In accord with this model, proteins involved in reduction of stomach acidity were found less expressed in *H. pylori* isolated from patients with AAG.

Urease B, a key enzyme for bacteria resistance to gastric acidity by catalyzing the hydrolysis of urea into ammonia and CO_2_, is an immunogenic protein: its epitope vaccination allowed a reduction in *H. pylori* colonization and inflammation of the gastric mucosa [42]. We hypothesized that an increase in UreB production in *H. pylori* from AAG patients compared with *H. pylori* from GC patients could be beneficial since it reduces gastric inflammation that is widely accepted to be related to GC pathogenesis. The importance of ammonia in *H. pylori* metabolism and virulence is underlined by the presence of several alternative routes for ammonia production, via enzymatic degradation of diverse amides and amino acids. Furthermore, network analyses with STRING showed that UreB protein are connected with the heat shock chaperone protein GroES (spot 227), which is known to induce a protective immunity against mucosal infection [43]. Both AAG and GC are known to be associated with a severe inflammatory response, that is associated with increased levels of reactive oxygen and nitrogen radicals around the colonizing *H. pylori*. In a previous proteomics study it was demonstrated that the infection with *H. pylori* strain 7.13 induces a severe inflammatory response in gerbils [11], that the authors associated with increased levels of reactive oxygen and nitrogen radicals at sites juxtaposed to colonizing organisms.

It is interesting to note that among the proteins highly decreased in AAG-isolated *H. pylori*, there was a flagellin A subunit (spot 42). This protein was known to polymerize together with flagellin B, and form the bacterial filaments, with an important role in both bacterial motility and virulence [13, 44–46].

A putative elongation factor-Tu was detected in *H. pylori* up-regulated proteome of both DU-*H. pylori* and GC-*H. pylori* (spots 63 and 89). The major role of this protein is to mediate the transfer of charged aminoacyl-tRNA to the A site of the ribosome during peptide elongation. In our *H. pylori* samples, this protein showed two isoforms with a different accumulation in relation to patient gastric disease.

In regards to the biological processes, proteins increasing in GC-*H. pylori* were mostly related to DNA processes (replication, transcription and translation). In particular, among the *H. pylori* proteins up-regulated in GC isolates we identified an elongation factor (spots 63 and 89), a DNA primase involved in RNA modification (spot 141), a DNA-directed RNA polymerase subunit α (spot 95), a DNA-binding protein HU (spot 234), a transcriptional regulator (spot 262), a 50S ribosomal protein L21 (spot 233), a ribosomal protein S12 methyltioltransferase (spot 232), and a 10 kDa chaperonin (spot 231) (Table 2; Fig. 4b). Interestingly, the DNA dependent RNA polymerase (RNAP) catalyzes the transcription of DNA into RNA, and it is composed of several subunits; the subunit α of RNAP has been identified among the proteins more specifically associated with gastric *H. pylori* species rather than enterohepatic ones [47]. Moreover, the C-terminal domain of the α subunit of RNAP, besides a primary role in the recruitment of RNA polymerase to various promoters, has a role in mediating interactions with several transcriptional regulators [48]. Concomitantly with these findings, Lin et al. [49] identified the subunit α of RNAP like a GC-related *H. pylori* antigen.

While the DNA primase encoded by the dnaG gene in an enzyme synthesizing short strands of RNA during DNA replication, and it is part of the replication machinery of the slowly growing *H. pylori* [50, 51]. Its presence may be related to a slow *H. pylori* growth related to the extremes of the human gastric environment. In addition, GC-*H. pylori* strains increased the content of a ribosomal protein. Xiao et al. [52] succeeded in classifying different *H. pylori* origins (P1 and P2) based on ribosomal proteins, which they estimated to represent the highest percentage (15%) of identified proteins. However, the differential up-regulation in GC-*H. pylori* strains may be only indicative of a higher demand of ribosomes, and, indirectly, a higher protein turnover as compared with the DU-*H. pylori* strains.

## Conclusion

We have successfully performed a DIGE comparative proteomics analysis of *H. pylori* strains isolated from patients affected by different gastric pathologies (AAG, DU or GC). Some of the identified proteins had not been characterized in gastric disease-related *H. pylori* strains before. The finding of differential protein profiles among *H. pylori* related groups confirms the difference in *H. pylori* strains in relation to gastric disease. In particular, in *H. pylori* isolated from DU-patients a higher content of proteins with antioxidant activity emerged (*aroQ*, *aspA*, *fldA, icd*, *oorA* and *scoB*), as well as an up-regulation of proteins belonging to metabolic pathways counteracting the high acid environment (*katA* and *napA*). While, in *H. pylori* isolated from AAG-patients there was a significant decrease in proteins neutralizing hydrogen concentrations through organic substance metabolic processes (*dnaG*, *tuf*, *trxB* and *groL*), underlying the different gastric environment of the two pathologies. In addition, a reduction of bacterial motility (*flhA*) was found to be associated with AAG-*H. pylori* isolates. In GC-*H. pylori* strains it emerged an increase in nucleic acid-binding proteins to be putatively involved in a higher demand of DNA- or protein-related processes. Some of the identified proteins may provide some new information in the understanding of the candidate mechanism(s) associated with the differential *H. pylori* behavior in human stomach disease(s), and indicate potential protein markers for the specific detection of DU *versus* GC-related *H. pylori*. Some of our identified proteins need to be further validated by functional analyses as well as at DNA transcriptional level, and it may be tempting to incorporate our protein expression data with those of *H. pylori* genomic works in order to get better insight into the differential *H. pylori* pathogenesis.

## Additional files

Additional file 1: Figure S1. List of peak masses enabling the identification of the neutrophil activating protein by mass spectrometry. The trypsin-digested peptides of the gel portion at ~ 15 kDa were also separated by MALDI-TOF to search for masses of the ‘neutrophil activating protein, NapA’. The list of peak masses, which were generated by an *in silico* tripsin-digestion of the protein P43313 corresponding to the NapA, are listed together with both those found in the spot 204 digestion, and those detected in the digested 15 kDa bands. (PPT 167 kb)

Additional file 2: Table S1. Protein pairs of the *Helicobacter pylori* strains isolated from patients affected by duodenal ulcer (DU) gastric cancer (GC) or autoimmune atrophic gastritis (AAG). Protein pairs from *H. pylori* cases were labelled with either Cy3 or Cy5 dyes and mixed with a Cy2-labelled internal standard, containing equal amounts of the all protein extracts. (DOCX 13 kb)

## Abbreviations

AAG: autoimmune atrophic gastritis
DU: duodenal ulcer
GC: gastric cancer
IARC: International Agency for Research on Cancer
IEF: isoelectric focusing
IPG: immobilized pH gradient
LC-MS/MS: liquid chromatography-tandem mass spectrometry
MALDI-TOF: matrix assisted laser desorption ionization-time of flight
PCA: principal component analysis
1DE: one dimensional electrophoresis
2DE: two dimensional electrophoresis
2D-DIGE: two-dimensional difference in gel electrophoresis
