# Supplementary figures and images for: Protein signature characterizing Helicobacter pylori strains of patients with autoimmune atrophic gastritis, duodenal ulcer and gastric cancer
Source: Infect Agent Cancer. 2017 Apr 27;12:22. doi: 10.1186/s13027-017-0133-x (PMC5408474; doi:10.1186/s13027-017-0133-x)

## Slide 1
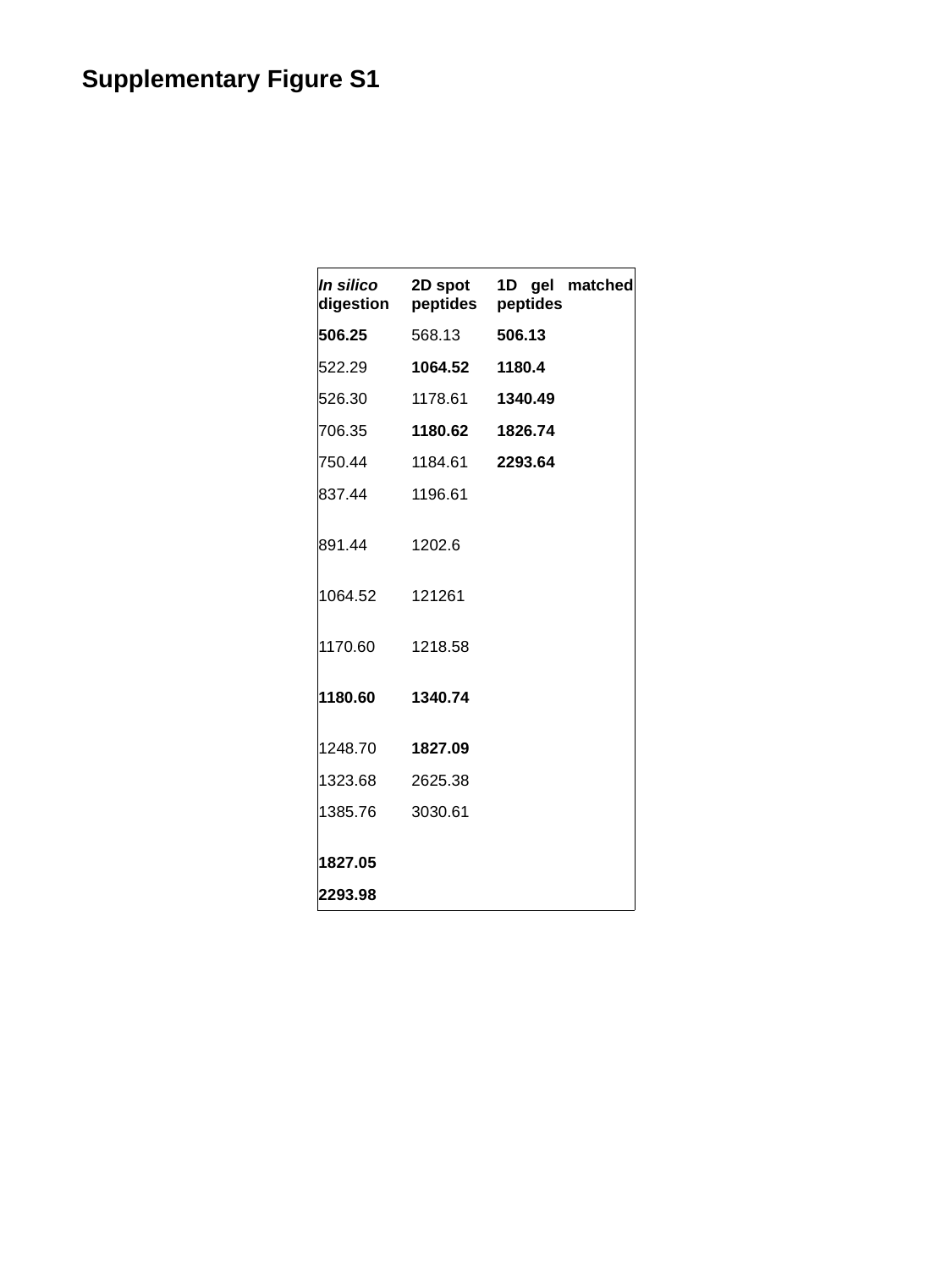

Supplementary Figure S1
| In silico digestion | 2D spot peptides | 1D gel matched peptides |
| --- | --- | --- |
| 506.25 | 568.13 | 506.13 |
| 522.29 | 1064.52 | 1180.4 |
| 526.30 | 1178.61 | 1340.49 |
| 706.35 | 1180.62 | 1826.74 |
| 750.44 | 1184.61 | 2293.64 |
| 837.44 | 1196.61 | |
| 891.44 | 1202.6 | |
| 1064.52 | 121261 | |
| 1170.60 | 1218.58 | |
| 1180.60 | 1340.74 | |
| 1248.70 | 1827.09 | |
| 1323.68 | 2625.38 | |
| 1385.76 | 3030.61 | |
| 1827.05 | | |
| 2293.98 | | |

Supplement: Supplementary file 1 — List of peak masses enabling the identification of the neutrophil activating protein by mass spectrometry. The trypsin-digested peptides of the gel portion at ~ 15 kDa were also separated by MALDI-TOF to search for masses of the ‘neutrophil activating protein, NapA’. The list of peak masses, which were generated by an in silico tripsin-digestion of the protein P43313 corresponding to the NapA, are listed together with both those found in the spot 204 digestion, and those detected in the digested 15 kDa bands. (PPT 167 kb) [file 13027_2017_133_MOESM1_ESM.ppt]
