# Supplementary material for: Protein signature characterizing Helicobacter pylori strains of patients with autoimmune atrophic gastritis, duodenal ulcer and gastric cancer
Source: Infect Agent Cancer. 2017 Apr 27;12:22. doi: 10.1186/s13027-017-0133-x (PMC5408474; doi:10.1186/s13027-017-0133-x)
Supplement: Supplementary file 2 — Protein pairs of the Helicobacter pylori strains isolated from patients affected by duodenal ulcer (DU) gastric cancer (GC) or autoimmune atrophic gastritis (AAG). Protein pairs from H. pylori cases were labelled with either Cy3 or Cy5 dyes and mixed with a Cy2-labelled internal standard, containing equal amounts of the all protein extracts. (DOCX 13 kb) [file 13027_2017_133_MOESM2_ESM.docx]

***Additional file 2: Table S1.*** *Protein pairs of the* Helicobacter pylori *strains isolated from patients affected by duodenal ulcer (DU) gastric cancer (GC) or autoimmune atrophic gastritis (AAG). Protein pairs from* H. pylori *cases were labelled with either Cy3 or Cy5 dyes and mixed with a Cy2-labelled internal standard, containing equal amounts of the all protein extracts.*

| ***Protein extract nr.*** | ***Patient nr.*** | ***Disease^(a)^*** | ***Localization^(b)^*** | ***Age*** | ***Sex^(c)^*** |
| --- | --- | --- | --- | --- | --- |
| *1* | *1* | *DU* | *A* | *37* | *F* |
| *2* | *2* | *DU* | *A* | *48* | *M* |
| *3* | *3* | *DU* | *A* | *42* | *F* |
| *4* | *4* | *DU* | *A* | *62* | *M* |
| *5* | *5* | *DU* | *A* | *44* | *M* |
| *6* | *6* | *DU* | *A* | *62* | *M* |
| *7* | *7* | *DU* | *C* | *52* | *F* |
| *8* | *8* | *DU* | *C* | *55* | *M* |
| *9* | *2* | *DU* | *C* | *48* | *M* |
| *10* | *3* | *DU* | *C* | *42* | *F* |
| *11* | *4* | *DU* | *C* | *62* | *M* |
| *12* | *9* | *GC* | *A* | *71* | *F* |
| *13* | *10* | *GC* | *A* | *67* | *M* |
| *14* | *11* | *GC* | *A* | *64* | *M* |
| *15* | *12* | *GC* | *A* | *74* | *M* |
| *16* | *13* | *GC* | *A* | *56* | *M* |
| *17* | *14* | *GC* | *A* | *40* | *M* |
| *18* | *15* | *GC* | *A* | *68* | *M* |
| *19* | *16* | *GC* | *A* | *61* | *F* |
| *20* | *17* | *GC* | *A* | *61* | *F* |
| *21* | *18* | *GC* | *A* | *85* | *F* |
| *22* | *19* | *GC* | *A* | *56* | *M* |
| *23* | *20* | *GC* | *A* | *46* | *F* |
| *24* | *21* | *GC* | *A* | *71* | *F* |
| *25* | *20* | *GC* | *C* | *46* | *F* |
| *26* | *12* | *GC* | *C* | *74* | *M* |
| *27* | *15* | *GC* | *C* | *68* | *M* |
| *28* | *18* | *GC* | *C* | *85* | *F* |
| *29* | *19* | *GC* | *C* | *56* | *M* |
| *30* | *21* | *GC* | *C* | *71* | *F* |
| *31* | *22* | *GC* | *A* | *73* | *M* |
| *32* | *23* | *GC* | *A* | *66* | *F* |
| *33* | *24* | *GC* | *A* | *66* | *F* |
| *34* | *25* | *GC* | *A* | *50* | *M* |
| *35*  *36* | *26*  *27* | *GC*  *GC* | *A*  *A* | *43*  *68* | *F*  *M* |
| *37*  *38*  *39*  *40*  *41* | *28*  *29*  *30*  *31*  *29* | *AAG*  *AAG*  *AAG*  *AAG*  *AAG* | *A*  *A*  *C*  *C*  *C* | *70*  *40*  *42*  *37*  *40* | *F*  *F*  *F*  *F*  *F* |

*^(a)^AAG, autoimmune atrophic gastritis; DU, duodenal ulcer; GC, gastric cancer; ^(b)^A, antrum; C, corpus; ^(c)^F, female; M, male.*
